# Research on the spatial correlation and formation mechanism between traditional villages and rural tourism

**DOI:** 10.1038/s41598-023-35486-w

**Published:** 2023-05-22

**Authors:** ZiYang Li, MeiYu Yang, XianLan Zhou, ZhiGang Li, HaiDong Li, FeiFei Zhai, Yan Zhang, YunXing Zhang

**Affiliations:** 1 School of Architectural and Artistic Design, Henan Polytechnic University, Jiaozuo, 454000 China; 2 School of Liberal Arts and Law, Henan Polytechnic University, Jiaozuo, 454000 China; 3 Jiyang College, Zhejiang A&F University, Zhuji, 311800 China

**Keywords:** Sustainability, Environmental impact

## Abstract

In recent years, the survival and development of traditional villages in China have been serious challenges. Rural tourism is regarded as an important way to solve rural problems, and the combination of rural culture and tourism has become a new power point for rural development. Therefore, it is necessary to explore the spatial distribution structure between traditional villages and rural tourism. In this paper, rural tourism was represented by the rural tourism characteristic village (RTCV), and Henan Province, China, was taken as a study area to analyze the distribution pattern and spatial correlation of rural tourism and traditional village (TV) and discuss the relationship between the spatial correlation and regional natural environment and socioeconomic factors. The results show that the coupling of the spatial correlation between RTCVs and TVs in Henan was clear. They could be divided into 5 regions based on geographical factors. In addition, the research summarized 4 typical spatial structures between TVs and RTCVs in Henan based on the regional symbiosis theory, and the spatial pattern formation mechanism of TVs and RTCVs was discussed based on three driving mechanisms. The spatial structure of the two can provide reference value for other developing countries and regions to achieve sustainable rural development.

## Introduction

Traditional villages (TVs) are an important component of the country space system. It is not only an important carrier of regional traditional culture and folk customs but also carries many historical memories, humanistic ecology, architectural aesthetics and social development tracks, known as the “living fossil” of the countryside^1–23^. However, with the acceleration of urbanization, traditional villages have been facing severe challenges, and revitalization work has become extremely urgent^4^. At the same time, rural tourism is regarded as an important way to solve rural problems, which promotes handicraft production in the agricultural sector, improves living conditions and protects cultural heritage and folk customs by using local natural resources and human resources^5,6^. It has been on the agenda of many local, regional and national policy makers^7^ and promoted as a tool to enhance the versatility of rural areas^8,9^. On the other hand, as a countryside with a symbiotic relationship with tourism^10^, the diversity and uniqueness of rural culture provide rich resources for the development of rural tourism. Rural culture can form a unique tourism resource for the tourism industry by developing the ancient architectural culture of the village into a rural characteristic landscape. The combination of rural culture and tourism has become a new power point in the countryside^11^. Therefore, it is of great significance to explore the spatial structure type between traditional villages and rural tourism to achieve the sustainable development of rural tourism and the revitalization of traditional villages.

Currently, there is a lack of consensus on the definition of rural tourism^12,13^. In this paper, rural tourism is represented by the rural tourism destination—rural tourism characteristic village (RTCV), which is the product of the Henan provincial government implementing the national rural revitalization strategy, with a total of 471^14^. Relevant studies have shown that traditional village and rural tourism destinations are regions interwoven with economic and social activities and have obvious comprehensive and regional characteristics^15,16^. Therefore, from the perspective of regional symbiosis theory, exploring the spatial structure of traditional villages and rural tourism destinations is necessary. The concept of symbiosis was first proposed and used in the field of biology in 1879^17^. After the 1950s, the idea of symbiosis penetrated many fields of society^18–20^. The symbiotic system includes three elements: the symbiotic unit, symbiotic mode, and symbiotic environment. Its core content is the symbiotic relationship composed of diverse symbiotic units^21^, which reveals the phenomenon of mutual cooperation, mutual benefit, coexistence and common development among units^22^. In China, with the deepening of research in recent years, an increasing number of scholars have applied symbiosis theory to ancient village tourism^23^, rural tourism^24,25^, cultural tourism integration^26^ and so on. As the decisive features in rural development^27^, symbiosis theory was used in the research on the mechanism of promoting the common prosperity of traditional villages, and rural tourism will be a meaningful practice. In this paper, the symbiotic unit specifically refers to the TVs and RTCVs. Symbiotic environments are external conditions for the analysis of symbiotic relationships between TVs and RTCVs, including natural, economic, and social environments. The symbiosis mode of traditional villages and rural tourism destinations will be analyzed from the geographical perspective.

Meanwhile, some spatial results on TVs and RTCVs have been obtained^28,29^. However, most studies are limited to the spatial distribution of TVs or RTCVs themselves^30,31^, studies on the correlation between the spatial distribution of ancient villages and tourism development elements^32^, and studies on rural tourism in typical TVs^33–35^. In addition, there are some similarities in the spatial distribution between historical villages and poverty-stricken villages^36^. Thus, research on the spatial structure and coupling of TV and RTCV can provide a clearer understanding of the economic situation in different rural areas, and a more precise rural development strategy will be implemented. The symbiotic spatial structure of the two can be applied to other developing countries and regions to provide an effective reference value for the sustainable development of rural tourism.

The research area of this paper is Henan Province, which is the largest agricultural province and one of the provinces with a large population in China^37^. In terms of population size, geographical area and economic output, it is comparable to medium-sized countries on the European continent, such as Sweden^38,39^. In addition, Henan has complex and diverse topography and is rich in tourist resources and cultural diversity, with a similar spatial distribution of traditional villages and rural tourism destinations^40,41^. This paper aims, first, to study the spatial distribution modes of TVs and RTCVs and to explore the relationships between the spatial distribution characteristics of villages and the multidimensional impact factors at the provincial level. Furthermore, their kernel density coupling relationship at the township level was revealed using spatial correlation. Finally, according to the analysis results, the spatial structure of rural tourism characteristic villages and traditional villages are discussed based on the theory of regional symbiosis. The research results can provide a reference for the sustainable development of traditional villages and rural tourism.

## Materials and methods

### Study area

**Figure 1:**
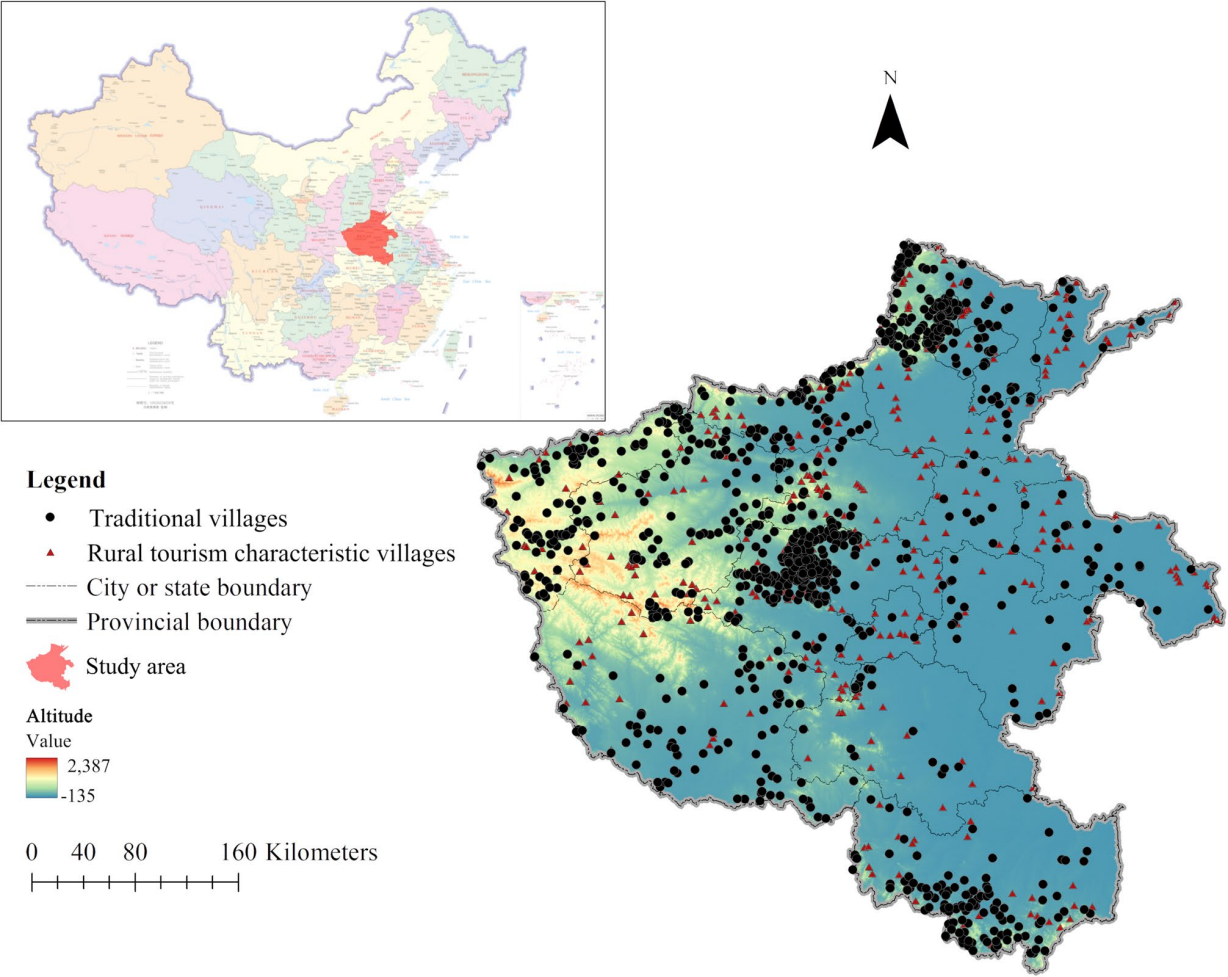
Spatial distribution of TVs and RTCVs in Henan Province, China. *Note* The basic map of China is derived from the standard map service website (http://bzdt.ch.mnr.gov.cn) with approval number GS(2022) 4316 produced by the Ministry of Natural Resources of the People’s Republic of China, and the base map has not been modified. A spatial distribution map of TVs and RTCVs in Henan Province was created by the authors using QGIS 3.30 (Geographic Information System, Open Source Geospatial Foundation Project. http://www.qgis.org/) based on digital elevation model (DEM) data from https://www.resdc.cn/.

Henan Province was named Yuzhou in ancient times and is known as ‘the hinterland of ancient China and the thoroughfare of ten provinces’. It is one of the important cradles of the Chinese nation and an important comprehensive transportation hub and information flow logistics center in China. Henan Province is bounded between 31° 23′ N–36° 22′ N and 110° 21′ E–116° 39′ E with a total area of 167,000 km^2^ and is in the transition area of the second and third steps in central China (Fig. 1). It is surrounded by mountains on three sides with high terrain in the west and low terrain in the east. The northern border is the Taihang Mountain region, the western border is the Funiu Mountains region, and the southern border is the Ta-pieh Mountains. The farming culture and geographical characteristics have created a fully preserved ancient village culture and rural tourism resources. The Henan Provincial government announced 6 batches of 1019 provincial-level TVs and 4 batches of 471 provincial-level RTCVs by 2022.

### Data sources

To study the spatial correlation between the distribution of TVs and RTCVs in Henan Province, this paper took 1019 TVs (see Supplementary Table S1 online) and 471 RTCVs (see Supplementary Table S2 online) as the objects identified by the Henan People’s Government. They were abstracted as points by calibrating longitude and latitude via the Baidu API. QGIS 3.30, GeoDa 1.20, and other analytical tools were used to analyze the role of environmental factors and social factors. Relevant data sources include (1) the topographic information of Henan Province DEM raster data with a resolution of 30 m and river data derived from the resource and environment data cloud platform of the Institute of Geographical Sciences and Resources, Chinese Academy of Sciences (https://www.resdc.cn/); (2) the administrative region (points) data were derived from the National Basic Geographic Information Center (http://www.ngcc.cn/); (3) the list of A-level scenic spots (see Supplementary Table S3 online) came from the Henan General Office of Culture and Tourism; and (4) data on population, per capita disposable income of all residents, road mileage and others were obtained from the national economic and social development statistical bulletin of relevant cities and prefectures in Henan Province in 2021 (the data, such as Henan Province 30 m DEM raster data, river system data, administrative region (points) data, population, per capita GDP data of county or city and road mileage. see Supplementary Data Collection D1 online).

### Methods and procedures

**Figure 2:**
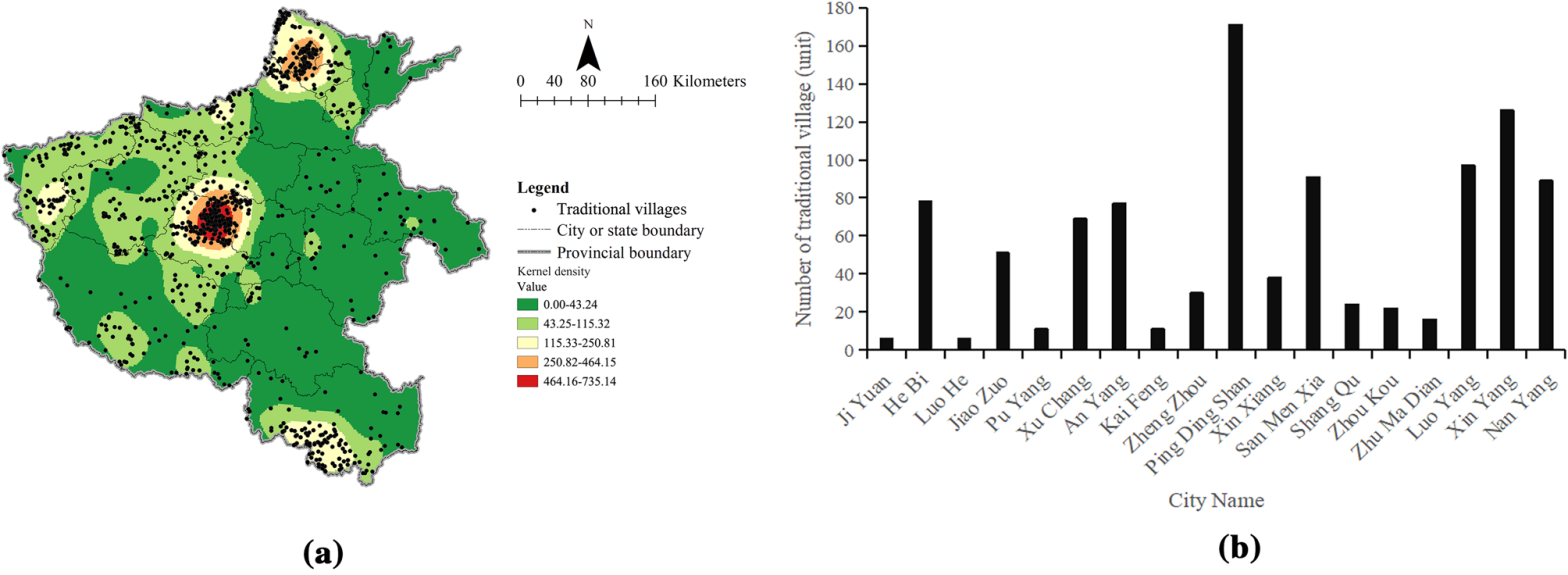
Distribution and kernel density of traditional villages in Henan Province (**a**) and quantity distribution diagram of traditional villages in various urban areas of Henan Province (**b**). Figure 2a was created with the Open Source software QGIS (version 3. 30—http://www.qgis.org/en/site/).

**Figure 3:**
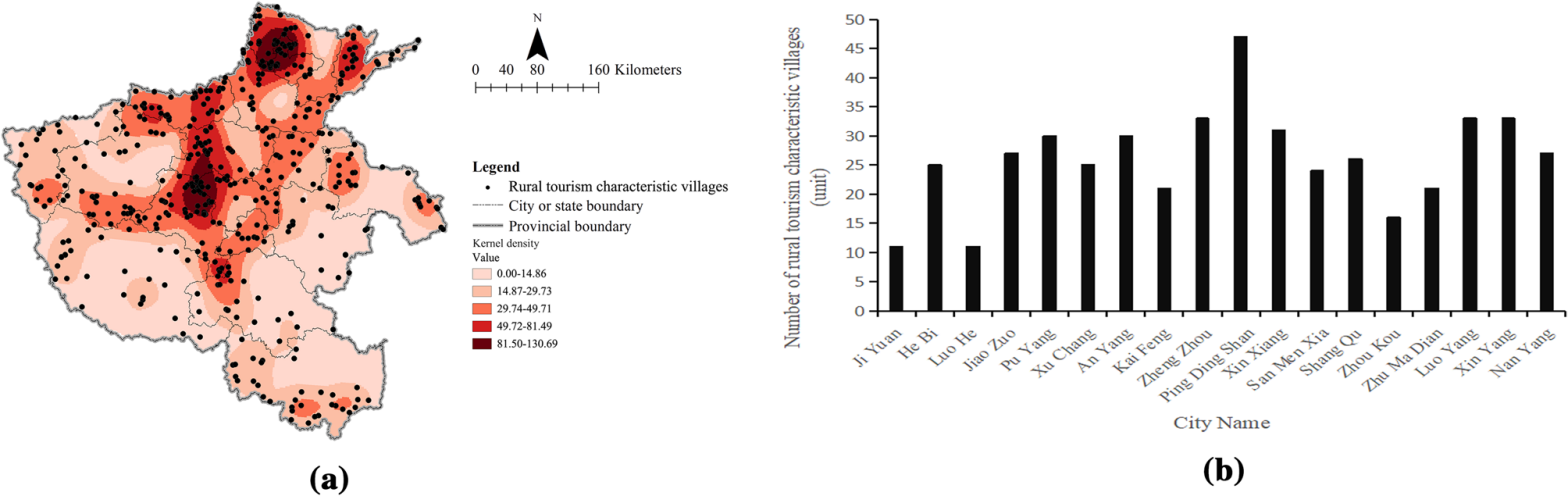
Distribution and kernel density of rural tourism characteristic villages in Henan Province (**a**) and quantity distribution diagram of rural tourism characteristic villages in various urban areas of Henan Province (**b**). Figure 3a was created with the Open Source software QGIS (version 3. 30—http://www.qgis.org/en/site/).

**Figure 4:**
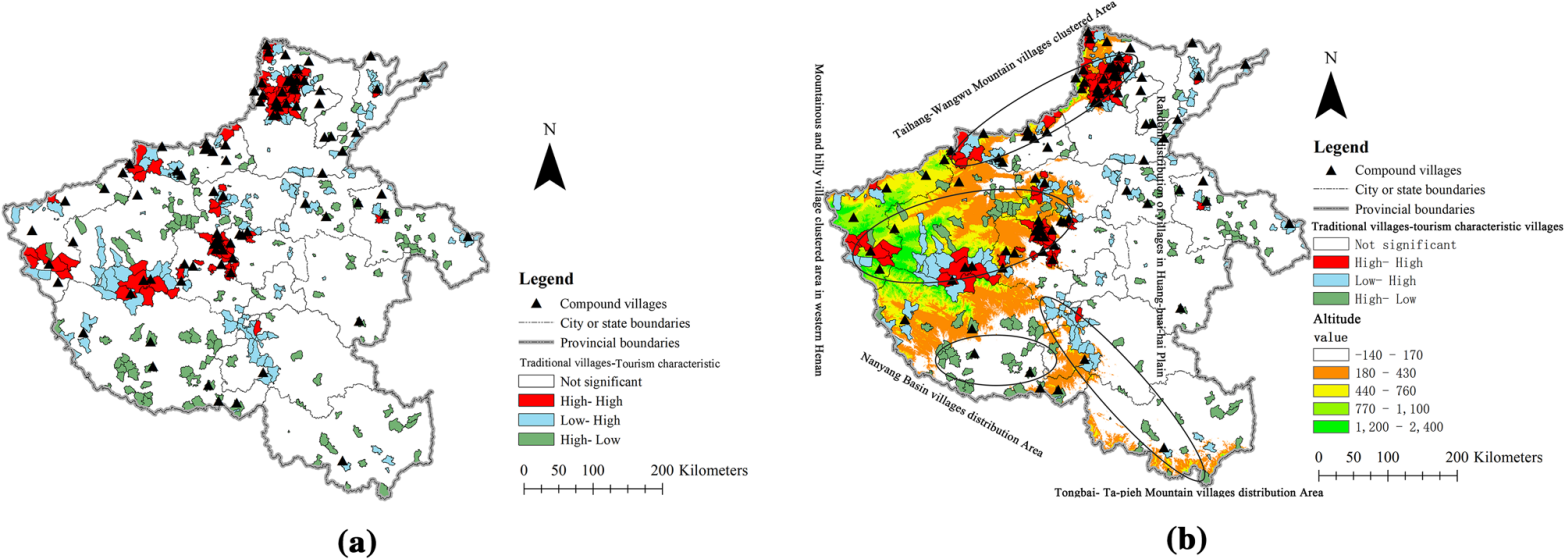
The LISA cluster diagram of the distribution of traditional villages and rural tourism characteristic villages (**a**) and the LISA cluster diagram of the distribution of traditional villages and rural tourism characteristic villages under topographic conditions (**b**). (**a**) and (**b**) were created with the Open Source software QGIS (version 3. 30—http://www.qgis.org/en/site/).

In this study, a series of spatial statistics and analysis techniques were applied to conduct a quantitative analysis of TVs and RTCVs in Henan. First, the longitude and latitude of TVs and RTCVs were obtained via the Baidu API, and data were entered and cleaned in Excel 2019 (Microsoft Corp, USA). Then, kernel density estimation and nearest neighbor index analysis based on QGIS 3.30 were employed to evaluate the spatial distribution of analysis points (Figs. 2a, 3a), and the quantity distribution diagrams of TVs and RTCVs in various urban areas were obtained through mathematical statistics in Excel 2019 (Figs. 2b, 3b). Finally, the spatial correlation relationship between the TV density index and the RTCV density index at the township level (Fig. 4) was analyzed by the bivariate spatial autocorrelation model based on GeoDa 1.20 (which is an open source, free software that can be used to analyze spatial statistics based on the spatial autocorrelation principle, download at http://geodacenter.github.io/). All layers were added to the WGS 84/UTM ZONE 51 N, EPSG code: 32651 Flat Coordinate Reference System.

**Figure 5:**
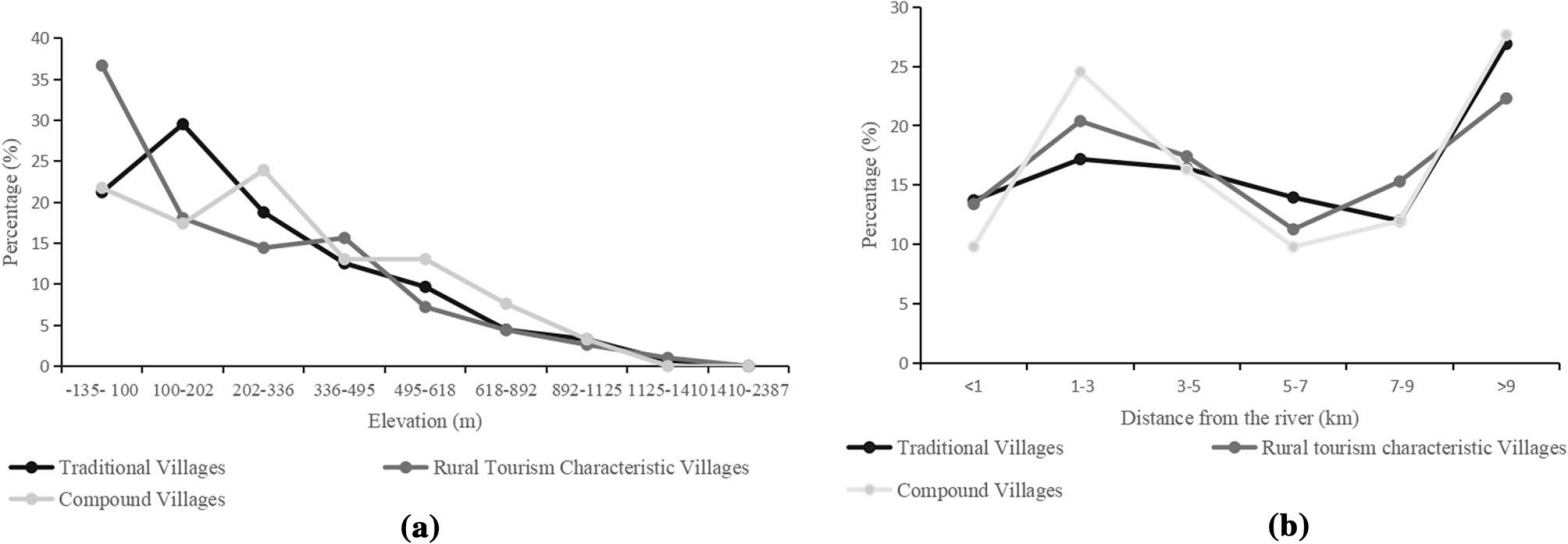
Distribution of villages around elevations in Henan Province (a) and distribution of villages around rivers in Henan Province (**b**).

**Figure 6:**
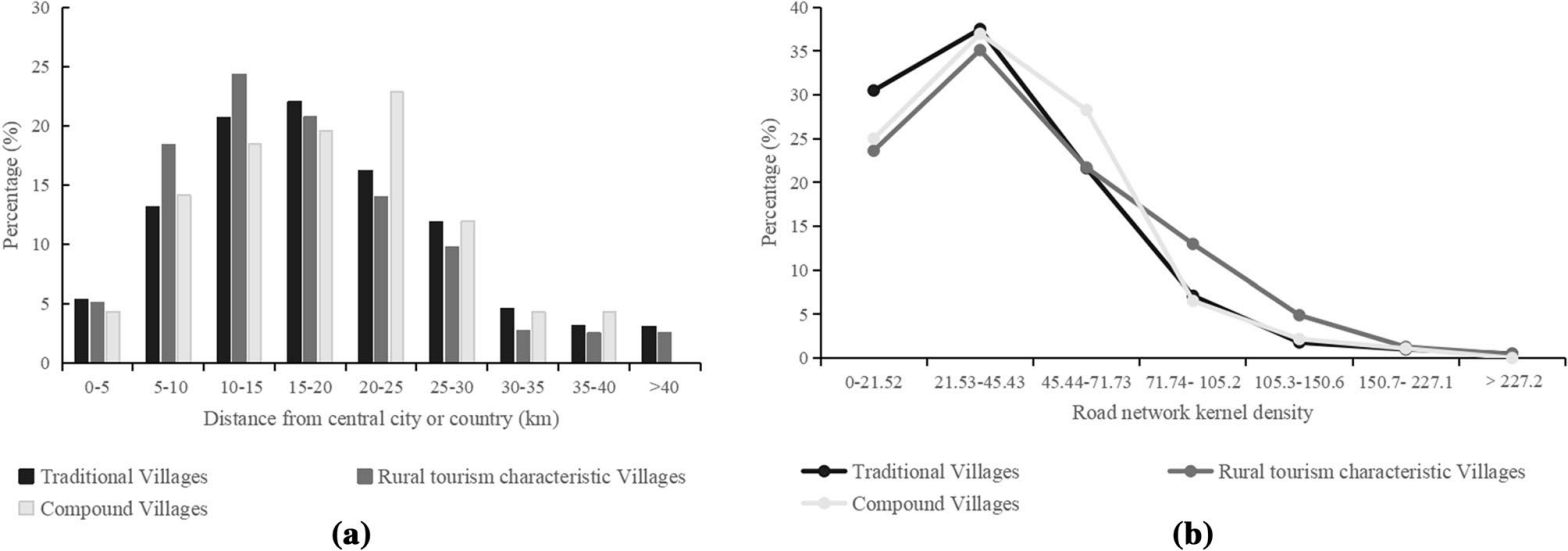
Distribution of villages around the central country or city in Henan Province (**a**) and distribution of villages around road network kernel density in Henan Province (**b**).

**Figure 7:**
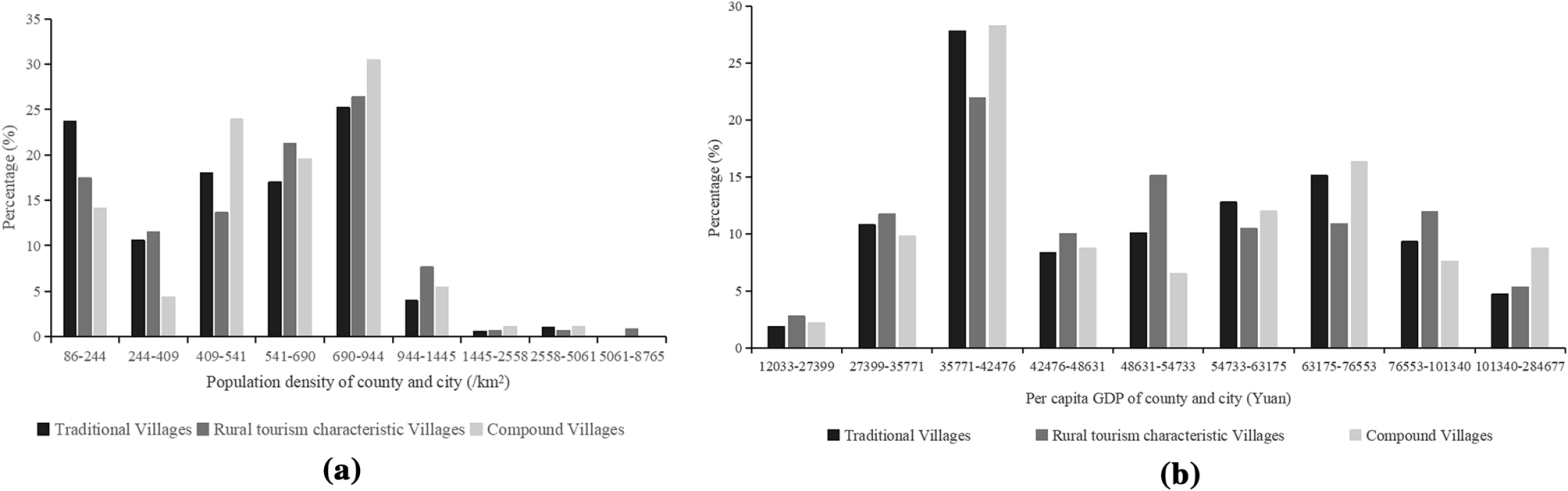
Distribution of villages around the population density of counties and cities in Henan Province (**a**) and distribution of villages around the per capita GDP of counties and cities in Henan Province (**b**).

**Figure 8:**
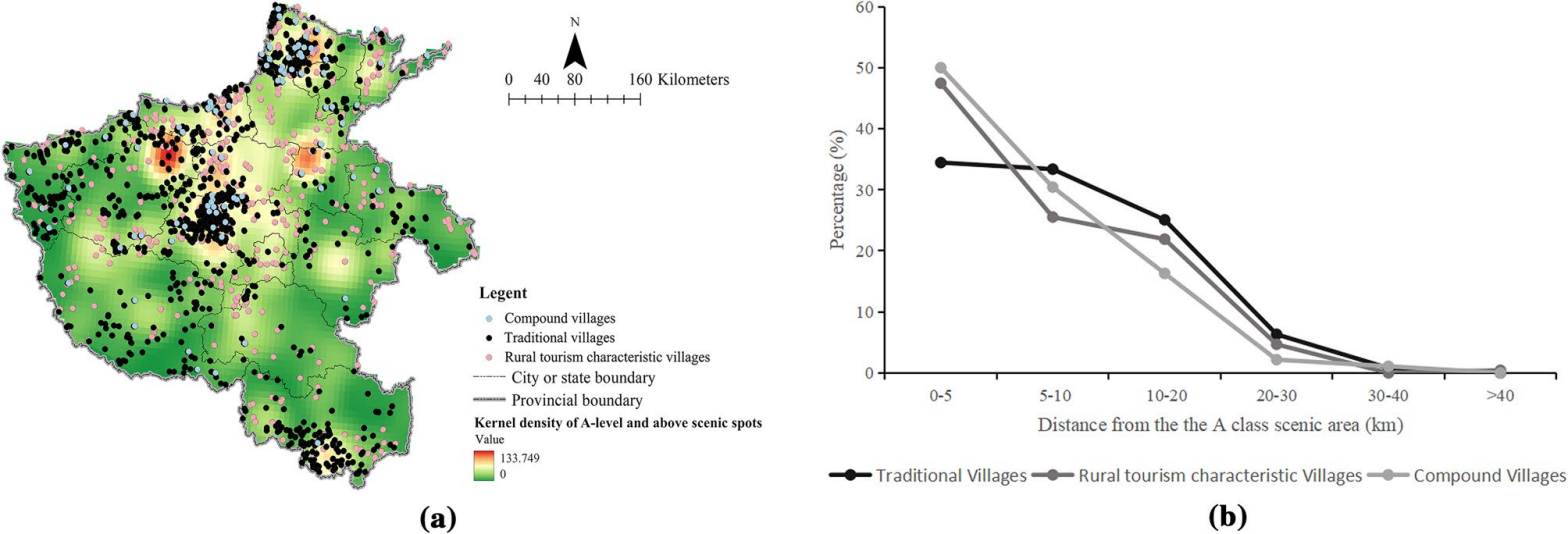
Spatial distribution of villages around the class A scenic area in Henan Province (**a**) and distribution of villages around class A scenic areas in Henan Province (**b**). (**a**) were created with the Open Source software QGIS (version 3. 30—http://www.qgis.org/en/site/).

Vectorized natural and socioeconomic factors. The DEM data were harnessed to derive slope in degree and topographic relief (the difference in elevation between the highest and lowest points in the area). The scope of river, city-county and scenic spot radiation was determined by buffer zone tools. Moreover, according to the data of the Seventh Population Census of China (http://www.stats.gov.cn/) and the Statistic Yearbooks of Henan of 2021 (https://tjj.henan.gov.cn/), using road length, population and economic income, road network density and per capita GDP were calculated for each county or city. All of the above factors were reclassified into different intervals based on the reclassification tool by table in QGIS. The number of TVs and RTCVs in different intervals of each influencing factor was counted by using count points in polygons, and the histogram and line chart were obtained by Excel 2019 mathematical statistics (Figs. 5, 6, 7 and 8b). The main methods of this study are as follows:

#### Kernel density estimation

To visualize the spatial dispersion and the location, shape and size of TV and RTCV aggregations, kernel density estimation can calculate the sample density distribution of the entire region according to the value of the input point elements and their distribution, and the result is a continuous raster diagram^42^. The expression is:

1 $$f\left( x \right) = \frac{1}{nh}\sum\nolimits_{i = 1}^{n} {k\left( {\frac{x - xi}{h}} \right)}$$

where f(x) is the kernel density estimation of villages, larger values indicate that the distribution of villages is dense; n is the number of villages; x- xi is the estimated distance from point x to sample xi; h > 0 is the bandwidth (search radius), users can set the appropriate values; and k is a spatial weight function.

#### Nearest neighbor index analysis

In geographical space, the nearest neighbor index can represent the proximity of point objects^43^. The villages were abstracted in the study area into a series of geographical points as follows: S1 = (X1, Y1), S2 = (X2, Y2), …, Sn = (Xn, Yn), where Sn is the observation event serial number^31^. Then, the index was used to analyse the global distribution pattern of TV and RTCV in Henan Province. The average nearest neighbor ratio in a region is:

2 $$R = \frac{{\overline{{r_{1} }} }}{{\overline{{r_{E} }} }}$$

where *R* is the nearest neighbor point index, $\overline{{r }_{1}}$ is an average value of the distance (*r*_1_) between the nearest points, and $\overline{{r }_{E}}$ is the nearest neighbor distance in theory. When *R* < 1, the village tends to be concentrated in space; when *R* > 1, the village is uniformly distributed in space; and when *R* = 1, the village is randomly distributed in space. The formula of $\overline{{r }_{E}}$ is:

3 $$\overline{r}_{E} = \frac{1}{2}\sqrt{\frac{n}{A}}$$

where *A* is the area value of the study area and *n* is the number of villages in the study area.

#### Spatial correlation analysis

Spatial correlation among geographically related data can be analyzed through spatial statistics^25^. In this paper, the bivariate local spatial autocorrelation method was used to reveal the kernel density coupling relationship between TVs and RTCVs in Henan Province, and a local indicator of spatial association (LISA) diagram was used to reflect the significance level of local spatial autocorrelation. The formula is:

4 $$Y_{\alpha \beta } = \frac{{X_{\alpha }^{i} - \overline{X}_{\alpha } }}{{\sigma_{\alpha } }}\sum\nolimits_{j = 1}^{n} {W_{{{\text{ij}}}} \frac{{X_{\beta }^{j} - \overline{X}_{\beta } }}{{\sigma_{\beta } }}}$$

where *α* = 1, 2… and *β* = 1, 2, …, *Yαβ* is the bivariate local spatial autocorrelation coefficient; ${\mathrm{X}}_{\mathrm{\alpha }}^{\mathrm{i}}$ is the value of the kernel density of the TVs in the township; ${X}_{\beta }^{j}$ is the value of the kernel density of the RTCVs in the township; $\overline{{X }_{\alpha }}$ is the average value of kernel density in TV, $\overline{{X }_{\beta }}$ is the average value of kernel density in RTCV; *δ*_*α*_*, δ*_*β*_ is the variance of *α, β*; and *Wij* is the spatial weight matrix between spatial units *i* and* j*.

## Results

### Spatial distribution of TVs and RTCVs in Henan Province

The nearest neighbor index of TVs in Henan was 0.539 by QGIS 3.30, indicating that the spatial distribution of TVs was clustered. Then, the spatial distribution of TVs was significantly different and tended to be clustered in space by kernel density analysis (Fig. 2a). At the provincial level, the distribution characteristics of TVs in Henan were as follows: the spatial distribution of TVs was denser in the northern, central and southern regions. According to the degree of aggregation of TVs, they were in the administrative junction of Hebi, Xinxiang, and Anyang cities in northern Henan, the junction area of Zhengzhou, Pingdingshan, and Xuchang cities in central Henan, and the southern area of Xinyang in southern Henan. The TVs in other cities were generally distributed in a low-density and discrete pattern. As shown in Fig. 2b, the top three cities with the largest number of TVs were Pingdingshan (171, 16.78%), Xinyang (126, 12.37%), and Luoyang (103, 10.11%). The common feature of the top three cities was that they have precious historical monuments and rich cultural heritage, such as Pingdingshan city^44^ and Xinyang city^45^.

The nearest neighboring index of RTCVs in Henan Province was 0.656, which demonstrates a cluster-type distribution in space. We analyzed the kernel density of RTCVs and found that the distribution of RTCVs in Henan Province was a multikernel clustering belt type (Fig. 3a). Similar to the distribution of TVs, RTCVs were mainly distributed in two linear regions and two core regions. The first linear distribution region was Anyang- Hebi- Xinxiang- Jiaozuo and Jiyuan cities; the second linear distribution area was Puyang- Xinxiang- Kaifeng- Xuchang- Luohe and Pingdingshan cities; and the other two concentrated distribution areas were the intersection of Xinxiang, Hebi, Anyang and the administrative junction of Pingdingshan and Xuchang. The average number of villages in each city was approximately 30 or less than 30 units, and the number of RTCVs in Pingdingshan ranked first, totaling 47 (Fig. 3b).

### Spatial correlations of TVs and RTCVs in Henan Province

Ninety-four compound villages (CVs, with characteristics of both traditional villages and tourism villages) were found by the spatial analysis function of QGIS 3.30 to overlap the spatial distribution of TVs with RTCVs, which accounted for 6.31% of the total village sample, 9.22% of all TVs and 19.50% of all RTCVs. This indicated that 1 out of every 10 TVs in Henan Province was a village with tourism characteristics, and 1 out of every 5 villages with tourism characteristics was a traditional village.

Subsequently, as shown in Fig. 4a, the red area indicates that the density of TVs and RTCVs was high in this township; the blue area indicates that the density of TVs was low and the density of RTCVs was high in this township; the green area indicates that the density of TVs and RTCVs was low, and the white area indicates no correlation between TVs and RTCVs. The results showed that the high-density cluster types of TVs and RTCVs (referred to as high-high, meaning the other two types are TVs in front and RTCVs in behind) were distributed at the borders of cities, such as Jiyuan and Luoyang and Nanyang and Pingdingshan. The low–high types were mainly concentrated at the border of Luoyang, Nanyang, and Zhumadian. The rest of the region was sparsely distributed; the high-low type presented a scattered distribution in 18 states, where the largest number was distributed in Luoyang, Nanyang, Xinyang, and Shangqiu. In Fig. 4b, high-high type areas were in the Ta-pieh-Tongbai Mountain area, and the mountainous and hilly clustered village area of western Henan was observed by observing the results spatially superimposed with the DEM from Henan Province. Nanyang Basin, with inconvenient transportation and a backward economy, is surrounded by mountains on three sides. There are many TVs and a few RTCVs, which form a gathering place of high-low types. In the Tongbai Mountain area, low–high type agglomeration areas formed, and Ta-pieh Mountain became an area mixed low–high types with high-low types. In the Huang-Huai-Hai Plain region, there are mainly high-low type and low–high type mixes. The most distributed areas are Pu yang, Kaifeng, and Shangqiu city. Due to the limitation of topography, the LISA of distribution shows that TVs and RTCVs cluster in mountainous and hilly areas, and patterns of villages tend to be random and scattered in the plain area.

## Factors for the spatial differentiation of TVs, RTCVs, and CVs in Henan Province

The spatial distribution of TVs and RTCVs is significantly influenced by the natural environment, the level of economic development, and social conditions^46–48^. In this study, we identified the factors that affected the distribution of TVs and RTCVs. Then, 9 factors were chosen, including 4 natural environmental factors and 5 socioeconomic factors. Finally, the spatial distribution characteristics of the villages were analyzed by mathematical statistics. The natural environmental factors were elevation, slope, topographic relief, and distance from the river. The socioeconomic factors were road network density, the population density of county and city (Population Data from the Seventh Chinese Census), per capita GDP in the county and city, distance from the county and city, and distance from the A-level scenic spots. The results were as follows:

### Elevation and topography

The geographic environment is one of the important factors affecting the distribution of villages, which not only provides development space for villages but also restricts the spatial layout of villages. Based on QGIS 3.30, the reclassification function was used to divide the raster map of the DEM from Henan Province into nine elevation bands: − 135 to 100, 100–202, 202–336, 336–495, 495–618, 618–892, 892–1125, 1125–1410, and 1410–2387; five slope ranges: 0–15, 15–30, 30–45, 45–60, and 60–75; and six topographic undulating height ranges: 0–15, 15–30, 30–45, 45–60, 60–75, and > 75. Mathematical statistical analysis methods were used to count the number of villages in each interval (Table 1). The results indicated that the three types of villages showed the same distribution pattern:

1. As shown in Fig. 5a, more than 60% of the villages were distributed under 300 m. With the rise in altitude, the number of villages was lower, which showed a downward trend. The number of TVs in each interval was 215, 299, 195, 127, 98, 45, 33, 7 and 0, accounting for 21.10%, 29.34%, 19.14%, 12.46%, 9.62%, 4.42%, 3.24%, 0.69% and 0%, respectively. The number of RTCVs in each interval was 183, 90, 72, 78, 36, 22, 13, 5 and 0, accounting for 36.67%, 18.04%, 14.43%, 15.63%, 7.21%, 4.41%, 2.61%, 1.00% and 0%, respectively. There were 60 CVs that had an elevation of under 300 m, accounting for 63.82%, and 34 CVs with an elevation between 300 and 1100 m, accounting for 36.17%.
2. More than 70% of villages were distributed under a 15° slope or 15 m topographic relief, and the whole situation shows a “△” (Table 1). In addition, as the slope or terrain fluctuation rises, fewer villages grow. There were 925 TVs, 395 RTCVs, and 70 CVs that had a slope of under 15°, accounting for 90.78%, 84.04%, and 74.47%, respectively. Moreover, there were 825 TVs, 330 RTCVs, and 59 CVs with a topographic undulating height below 15 m, accounting for 80.96%, 70.21%, and 62.77%, respectively. There was only 1 RTCV with a slope above 60°, the other types were 0, and there were 2 TVs and 3 RTCVs with terrain reliefs above 60 m.

**Table 1:** The villages in different zones of slope and topographic relief in Henan (unit).

Slope/(°)	Number of TVs	Number of RTCVs	Number of CVs	Topographic relief/(m)	Number of TVs	Number of RTCVs	Number of CVs
0–15	925	395	70	0–15	825	330	59
15–30	86	59	21	15–30	168	97	23
30–45	8	15	3	30–45	21	31	11
45–60	–	–	–	45–60	3	9	1
60–75	–	1	–	> 60	2	3	–

### Relationship with rivers

Water has been the source of life since the earliest human civilization was born along the river. In early agricultural society, people took the water source as an important reference condition for village site selection. In modern society, the river system is also an indispensable tourism resource. In this study, the rivers were abstracted into a series of geographical line elements, and parallel strip polygons were set up at 1 km, 3 km, 5 km, 7 km, 9 km, and > 9 km from the line element as the central axis. As shown in Fig. 5b, 30.87% of TVs, 33.76% of RTCVs, and 33.69% of CVs were distributed within 3 km of the river. The location selection of rivers was crucial to not only the formation of TVs but also the development of RTCVs and CVs. In addition, another interesting phenomenon was found: most villages were distributed in the intervals of > 9 km, accounting for 26.89%, 22.19%, and 27.65%, and the intervals of 1–3 km followed, accounting for 17.17%, 20.38%, and 24.46%.

According to the relevant literature review, the reason might be that Henan is the only province that crosses the Yellow River, Haihe River, Huaihe River, and Yangtze River systems in China. There are many rivers, and the flat terrain is fertile soil in this region, benefiting the development of agriculture and forming early human settlements. However, with human activities, the soil erosion of the Loess Plateau in the middle reaches of the Yellow River is serious and leads to serious flooding and frequent dike diversion^49^. As a result, low-altitude plain areas, such as Puyang, Shangqiu, Kaifeng, Zhoukou, and other areas, were affected by floods, which made people intentionally homeless. Therefore, ancient villages near rivers were not suitable for preservation, and most human settlements were far from rivers.

### Central county or city and road traffic

Thünen developed the theory of agricultural location and believed that geographical location had important guiding significance for agricultural production layout^50^. In modern society, the road traffic network is closely related to the regional economic development level and has become the booster for the transformation of traditional villages into modern villages^51^. In addition, road traffic is the channel between the tourism destination and the tourism source area^52^. In view of the above, without regard to the other obstacles, the main highway buffer zone was established using radii of 5 km, 10 km, 15 km, 20 km, 25 km, 30 km, 35 km, and 40 km and provided spatial overlay analysis for the villages and buffer zones. The road network density within the region was calculated by kernel density valuation. Then, spatial overlay analysis was used to superimpose the correlation between road network density and the distribution of villages. The specific results are shown in Fig. 6.

As shown in Fig. 6a, villages were mainly distributed in the 1 h highway traffic circle, concentrated 10–20 km away from the central countries and cities, which was beneficial for people to carry out tourism and leisure activities. The number of villages in each interval showed a trend of rising first and then decreasing significantly. The number of TVs peaked in the range of 15–20 km and then decreased step by step. However, RTCVs were slightly better than TVs, whose number peaked in the range of 10–15 km and then declined stage by stage. The peak of the number of CVs moved to the right, reached 20–25 km, and then declined. According to the above findings, the government and planners can formulate relevant strategies to promote the coordinated development of rural tourism and traditional villages and revitalize traditional villages in this range. In Fig. 6b, the number of villages gradually decreased with the increasing kernel density of the road network, and more than 60% were distributed in the low density of the road network. The peaks of TVs, RTCVs, and CVs were 21.53–45.43 and then decreased gradually. The Chinese government continues to optimize the regional road network to solve the ‘last kilometer’ problem in the countryside.

### Population and economy

As shown in the pictures, the TVs and RTCVs were mainly distributed in the interval of fewer than 944 people per 1000 square meters (Fig. 7a). The numbers of TV and RTCV reached peak values of 690–944 and then were 86–244 and 541–690, respectively. The numbers of TV and RTCV were the lowest in the interval of 244–409, approximately 10% and 5%, respectively. In terms of per capita GDP (Fig. 7b), the distribution quantity of the two types of villages was totally scattered. The number of villages distributed in each interval was generally low, less than 15%. The problem was that the abnormal value of village number distribution was concentrated in the range of 35,771–42,476, and the quantity percentage was similar, accounting for approximately 28%, whose reasons need to be further explored.

All above, the distribution of TVs and RTCVs had a certain synchronization in population and economy. Areas with higher per capita GDP or population generally had fewer TVs and RTCVs, which meant that the distribution of TVs and RTCVs had obvious characteristics of economic marginalization. The unification and coordination of the cultural and economic values of traditional villages played a vital role in the sustainable development of traditional villages^53^. Therefore, local governments should prosecute their duties effectively, develop relevant preferential treatment strategies, organically combine the protection of traditional village culture with regional economic development and poverty alleviation, attract young people to return to their hometowns to start businesses, and increase the speed of capital return. Maximize the cultural vitality and development potential of traditional villages.

### Scenic spot resources

Tourism resources are the material support of space competition in tourism regions^54^. A-level and above scenic spots not only have rich tourism resources and tourism attractions but also support the development of rural tourism areas in China. Their spatial distribution affects the spatial distribution of rural tourism areas^55^. Relying on favorable terrain and profound cultural deposits, there were 15 5A scenic spots, 190 4A scenic spots, 271 3A scenic spots, 103 2A scenic spots, and 1 A scenic spot, with a total of 580 A-level or above scenic spots in Henan Province.

The scenic spots were abstracted into a series of geographical points as the center, and the buffer was constructed as radii of 5 km, 10 km, 20 km, 30 km, and 40 km for the spatial overlay analysis. As Fig. 8 shows, the spatial distribution of TVs and RTCVs had the typical characteristics of depending on scenic spots; the farther the distance from scenic spots, the fewer the number of villages. In addition, most of the scenic spots were in the mountains and rivers, which coincided with the distribution area of traditional villages.

### Discussion of factors affecting spatial differentiation

In summary, from a mathematical-statistical perspective, the following were found:

1. With increasing elevation, topographic relief, and slope, the number of traditional villages and rural tourism characteristic villages constantly decreased.
2. Affected by the diversion of the Yellow River and human activities, more than one-fifth of the villages were distributed outside 9 km from the main river.
3. More than half of the villages were concentrated in the range of 5–25 km from the county or city, but as the density of the road network increased, the number of villages decreased.
4. More than 60% of the villages were distributed in areas with low population density, and the abnormal value of village number distribution was concentrated in the range of 35,771–42,476 (per capita GDP in the county and city).
5. The farther the distance from the scenic spots, the lower the distribution of villages, and the villages had a typical dependence on tourist attractions.

Among natural factors, different geographic carriers and regional spaces affect or restrict human activities^56^. Relevant studies have also shown that the spatial distribution of TVs and RTCVs was negatively correlated with altitude and elevation^28,41^. However, as shown in Fig. 5a, the percentage of traditional villages peaked in the elevation range of 100–202, indicating that in Henan, the elevation has a nonlinear relationship with the number of traditional villages, and it is not that the lower the altitude is, the more suitable it is for the development of traditional villages^57^. Historically, the eastern plain of Henan Province has been disturbed by the bursting of the Yellow River floods for a long time (there were 42 floods between 1901 and 1948 alone^49^). Thus, compared with the conclusion that most traditional villages are distributed along the river^58,59^, the spatial distribution of TVs in Henan Province should be analyzed specifically due to natural factors. Relatively speaking, as the product of modernization, the potential value of RTCVs was more likely to be stimulated by low-altitude plain areas and appropriate river distances.

For socioeconomic factors, backward economic and transportation conditions provide a favorable environment for the protection of TVs^56^. In contrast, an increase in income may cause conflicts of interest and an imbalance in “rights relations”, which will affect rural resources and further lead to a lack of “rurality” and hinder the development of rural tourism^60^. Thus, the distribution of TVs and RTCVs had obvious characteristics of economic marginalization. Furthermore, the scenic spots had a certain radiation effect on the surrounding area. The famous case was the symbiotic development between the ancient villages of Hui Zhou and Huangshan Mountain. According to a survey, 67.1% of tourists visited the Huangshan Scenic Area or plan to visit the Huangshan Scenic Area before visiting Xidi village^61^. Therefore, the coordinated development of rural tourism regions would be promoted through the unified and indivisible tourist destination image established in the minds of tourists by rational planning of the tourist routes of villages and scenic spots.

## Spatial structure and mechanism formation

### The spatial structure type between traditional villages and rural tourism characteristic villages

**Figure 9:**
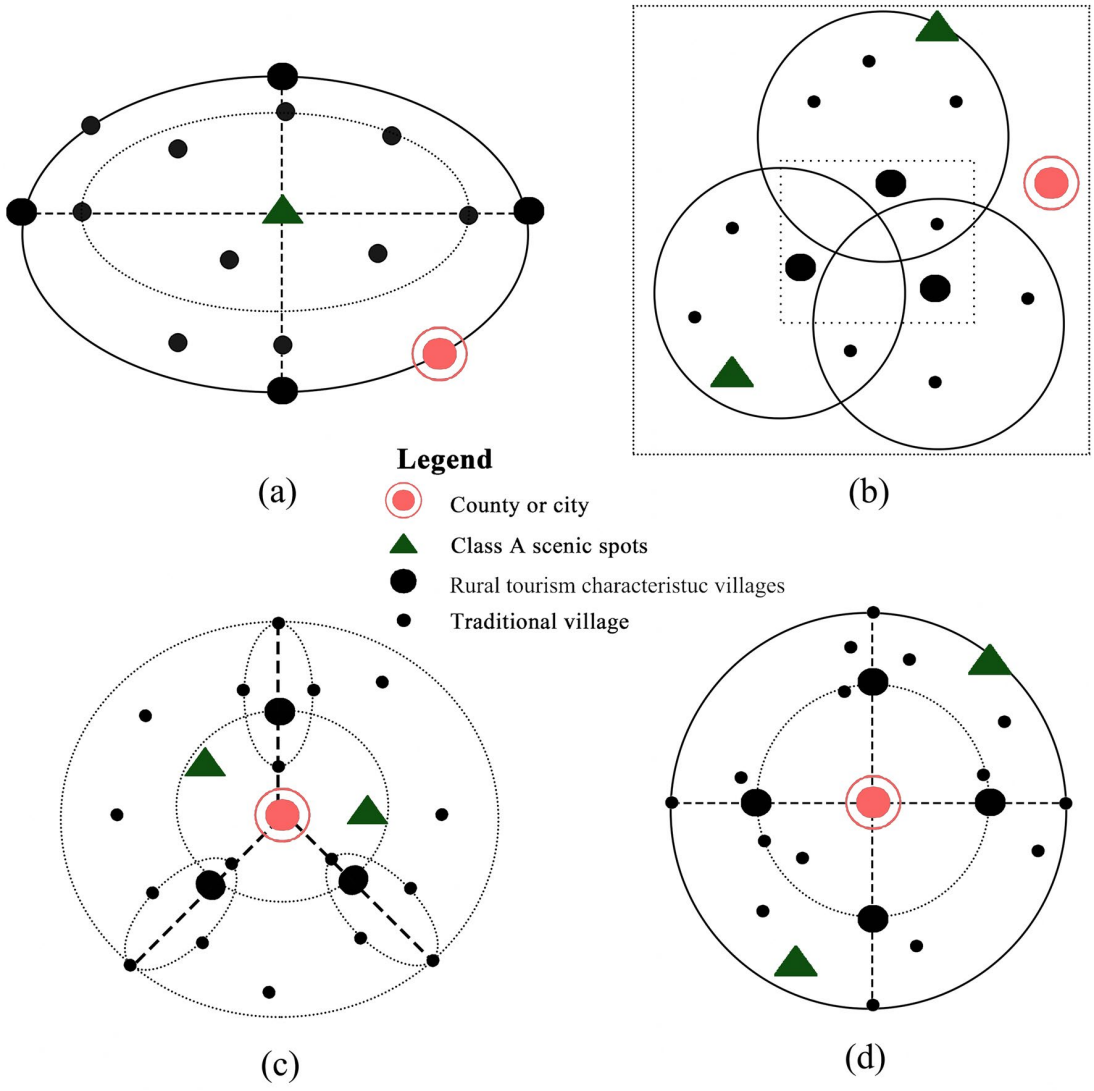
The spatial structure type between TVs and RTCVs.

The symbiotic model is the way symbiotic units interact and is also the combination of the symbiotic organizational model with the behavior pattern^62^. According to the degree of organization, symbiosis can be divided into four models: point symbiosis, intermittent symbiosis, continuous symbiosis and integrated symbiosis. According to the behavior model, it can be divided into four models: parasitic, partial symbiosis, asymmetry and symmetric reciprocal symbiosis. In this part, the spatial distribution structure with the integrated and mutually beneficial symbiotic model between TVs and RTCVs could be classified into 4 types according to the spatial pattern characteristics of each geographical region (Fig. 9): (1) natural attractions as the center, RTCVs as the nodes and activation of the TVs, (2) multicore central RTCV place distribution mode, (3) integrated corridor of RTCVs and TVs, and (4) central city distribution mode.

1. Natural attractions as the center, RTCVs as the nodes and activated TVs
  TVs and RTCVs in Henan Province were markedly affected by the center effect of scenic spots, most of which were in the transitional zone between plains and mountainous areas and distributed in clusters. Thus, the natural attractions were taken as the center to make RTCVs the nodes under the mountain natural attractions to influence several TVs on the plains. However, due to terrain obstructions and limits, RTCVs have difficulty affecting traditional villages at the depths of mountains. The patterns of village distribution presented as an elliptical circle, where TVs radially surrounded the RTCVs. This structure was suitable for mountainous and hilly areas with complex terrain (Fig. 9a).
2. Multicore central RTCV place distribution mode
  This structure was common in the low elevation area of the plain (Fig. 9b). The spatial distribution of rural settlements is usually dispersed on plains or in low-altitude hilly regions^16,63^. In these areas, two or more RTCVs combine to form a multicore center to integrate their market resources for TVs. Owing to ancient residential buildings, the natural environment, human activities and relics, unique cultural connotations, and external expression with regional style and art in traditional villages, the comprehensive landscape complex fed tourism feature villages and avoided RTCVs with ‘thousands of villages with the same features’.
3. Integrated corridor of RTCVs and TVs
  As Fig. 6 shows, most of the RTCVs and TVs were distributed in low traffic network density areas. RTCV and TV locations and transportation advantages should be used to form a spatial pattern along the axes of roads. Central cities or class A scenic spots were located in the center of the distribution pattern, where TVs and RTCVs were distributed along the axes of roads to form an integrated corridor. The RTCVs formed the connecting link in these corridors, where they could reach the maximum service radius (Fig. 9c).
4. Central city distribution mode
  As shown in Fig. 6, the distribution of rural settlements was intensive around cities. The number of RTCVs was maximum in the range of 10–15 km away from the cities. According to central place theory, RTCVs core service areas around the city were established, while the spatial organization structure of cities, RTCVs, and TVs should be optimized and reorganized (Fig. 9d). The city center provided balanced basic services to the surrounding TVs through RTCVs. RTCVs and TVs could receive all kinds of tourists from cities and vice versa, which could achieve win‒win results.

### Spatial pattern formation mechanism between traditional villages and rural tourism characteristic villages

China has a long history of farming-reading culture. Thus, traditional villages took shape very early. They are rural settlements spontaneously formed in the midst of long-term interactions between mankind and nature^64^. With the rapid process of economic and social development and urbanization, the survival and development of traditional villages have been restricted and challenged. However, with the proposal of China’s traditional village protection policy and the implementation of the rural revitalization strategy, traditional villages have been reborn and revitalized by relying on unique cultural and humanistic values. At the same time, rural tourism is the product of the modern economy, which originated in the Industrial Revolution and flourished by taking advantage of unique natural conditions. Although constrained by social conditions such as the economy and transportation, it still develops steadily with the support of national policies. In summary, the key impact mechanism of the spatial pattern of traditional villages and tourist villages in Henan Province can be refined into 3 categories: basic bearing mechanism, interventional restraint mechanism, and catalytic growth promoting mechanism. The formation and development of the spatial pattern of traditional villages and tourist villages was the result of the joint action and interaction of three driving mechanisms.

1. *Basic bearing mechanisms*, including natural factors such as elevation, terrain and rivers, provide a supportive material basis for the formation of villages, such as land and water resources, and provide a strong endogenous power for rural tourism. According to the previous analysis, the number of traditional villages and rural tourism characteristic villages decreases with the increase of altitude, and the farther away from the river, the fewer the number was; both were concentrated in the three mountain areas of Henan Province: Taihang-Wangwu Mountain Area in North Henan, Funiu-WaiFangshan Mountain Area in Western Henan, Ta-pieh-Tongbai Mountain area in North Henan. These areas provide fertile soil, clean water and natural defense barriers for the formation of villages and provide favorable conditions for the formation and development of the spatial correlation between traditional villages and rural tourism featured villages.
2. *Interventional restraint mechanisms*, including the economic development level, distance from cities, GDP per capita and other economic factors. Generally, the lagging level of social and economic development limits urban and rural construction in the region and maintains a stable and coordinated human-land relationship to a certain extent, which is conducive to the preservation of traditional villages and promotes the spatial distribution clusters of villages. The traditional villages in Henan Province are clustered in mountainous areas, far away from the urban center, with low population density, backward economic conditions and inconvenient transportation, which are conducive to the preservation of villages. However, many traditional villages are located in the urban radiation area, and due to the in-depth promotion of new urbanization, the local style and pattern of villages with agricultural civilization have been destroyed, and a situation of “one out of ten villages survived” has emerged. In contrast, convenient transportation and proper distance from the city promote the rapid development of rural tourism, and limited economic conditions and weak infrastructure restrict the development of rural tourism.
3. *Catalytic growth promoting mechanisms*, including social factors such as cultural history and policies. From the perspective of physical geography, natural, cultural, economic and other leading factors are integrated, and the principle of multifactor influence priority is adopted. Based on the administrative division unit and taking traditional villages as the research object, Henan’s cultural regions were divided into 5 categories: Tianzhong Cultural Area in Central Henan, Huanghuai Cultural Area in Eastern Henan, Heluo Cultural Area in Western Henan, Chu Cultural Area in Southern Henan, and Henei Cultural Area in Northern Henan (Table 2)^65,66^. Cultural diversity has created different types of traditional villages, and the uniqueness of the landscape has also prompted the adoption of different tourism policies. At the same time, to implement the national rural revitalization strategy, the Henan government accelerated the development of rural tourism through cultural and natural landscape resources^67^. With the support of government policies, the sustainable development of traditional villages and rural tourism can be realized by combining rural characteristic culture with tourism.

**Table 2:** Five cultural divisions in Henan Province (Table Drawing of Reference^61^).

Culture area	Regional	Feature	Strategy
Henei cultural area in north Henan	Taking Jiaozuo as the core to influence Xinxiang and other places, the villages were located in the southern Taihang Mountains	Traditional villages with strong regional characteristics built of stone	Regional tourism boutique routes have been developed by using the magnificent landscape of Taihang- Wangwu Mountain
Song mountain cultural area in central Henan	The villages were distributed in the area around Songshan Mountain and concentrated at the administrative junction of Baofeng, Jiaxian and Yuzhou	One of the core regions of the origin of Chinese civilization and cultural integration^68^; Located at the intersection of east and west, north and south in Henan, there are many traditional villages^69^	Relying on the regional advantages of Zhengzhou and Luoyang, the high-quality tourism cultural circle around Songshan Mountain will be build
Heluo cultural area in west Henan	The geographical scope is centered on Luoyang, including the area of western Henan and southern Shanxi, which is one of the most abundant topographical features in Henan	The Core culture of regional culture in ancient Chinese history, also be the “root” of the Chinese culture^70^	Rely on characteristic dwellings, such as “Below Grade Courtyard Style Subterranean”, the rural household service was provided
Chu (Hubei) culture area in southwest Henan	It is located in Nanyang Basin surrounded by mountains on three sides and low in middle and high schools around, with typical landscape pattern^71^	The cultural characteristics of openness, integrating the cultural essence of Shaanxi, Henan and Hubei	Using the landscape pattern of Xinyang City, build a “landscape city”
Tianzhong cultural area in south Henan	Including Xinyang City and Zhumadian City, the whole area was composed of plains, shallow mountains, mountains and other landforms	The transitional zone between the northern and southern cultures of China,which is under the comprehensive influence of Tianzhong Culture and Chu(Hubei) Culture^72^	Relying on the red education and ecological resources of Ta-pieh- Tongbai Mountain, “red tourism” has been developed

## Conclusions

With the development of the social economy, rural tourism has become an important way to promote the development of traditional villages. In this paper, traditional villages and rural tourism characteristic villages in Henan Province, China, were taken as the research object. GIS analysis methods were used to identify the spatial distribution between TVs and RTCVs. The results show the following: First, the spatial distributions of TVs and RTCVs were similar in Henan Province. They were mainly distributed in three high-density clusters where the terrain was dominated by mountainous and hilly areas: the administrative junction of Hebi, Xinxiang, and Anyang cities in northern Henan Province, the junction area of Zhengzhou, Pingdingshan, and Xuchang cities in the midland of Henan Province, and the southern area of Xinyang in southern Henan Province. Second, the city with the largest number of tourism feature villages and traditional villages was Pingdingshan, followed by Xinyang and Luoyang. These cities had abundant tourism resources and numerous historical monuments in common. Third, the spatial correlation relationship between the traditional village density index and the tourism feature village density index was obvious at the township level. Thus, the spatial distribution of village correlations was divided into 5 regions: Taihang-Wangwu Mountain village clustered area, mountainous and hilly village clustered area in western Henan Province, Nanyang Basin village distribution area, Tongbai-Ta-pieh Mountain village distribution area, and random distribution of villages in the Hang-Huai-Hai Plain.

Then, based on the driving factor correlation analysis and mathematical statistics of TVs and RTCVs, four typical mutually beneficial and symbiotic spatial structures between TVs and RTCVs were summarized from a geographical perspective. They were (1) natural attractions as the center, RTCVs as the nodes to activate the TVs, (2) multicore central RTCV place distribution mode, (3) the integrated corridor of RTCVs and TVs, and (4) central city distribution mode.

Finally, combining natural, economic and social factors, the spatial pattern formation mechanism of TVs and RTCVs was discussed based on three driving mechanisms: the basic bearing mechanism, the intervention constraint mechanism, and the catalytic growth promoting mechanisms.

## Supplementary Information

Supplementary Information 1.

Supplementary Table S1.

Supplementary Table S2.

Supplementary Table S3.

## Data Availability

All data generated or analysed during this study are included in this published article [and its supplementary information files].
